# Social media data and its potential for pharmacovigilance: a comparative analysis of reported prevalences regarding drug-induced gingival overgrowth (DIGO) 

**DOI:** 10.1007/s00210-026-04983-w

**Published:** 2026-01-23

**Authors:** Philipp Friedrich Georg Ried, Roland Seifert

**Affiliations:** Institute of Pharmacology, Hannover Medical School, Carl-Neuberg-Str. 1, D-30625 Hannover, Germany

**Keywords:** Drug-induced gingival overgrowth, DIGO, Calcium channel blockers, Immunosuppressants, Anticonvulsants, Swelling of the gums, Adverse drug reaction, Online survey, Dentistry

## Abstract

Drug-induced gingival overgrowth (DIGO) is an increasingly relevant topic among dentists, physicians and patients as well. Due to high prescription rates of calcium channel blockers, anticonvulsants and immunosuppressants, the prevalences of this adverse effect should not be underestimated. To identify differences in the prevalence rates presented, but also possible similarities in the available data, we compared information from package leaflets, social media platforms, current literature and summaries of product characteristics. To obtain a comprehensive overview of the current situation regarding DIGO, various sources were used for data collection. The social media platforms Facebook, YouTube, X, TikTok and Instagram were examined using various search terms, and the posts were then evaluated. Triggers of DIGO, treatment strategies, prevalences and the country of origin of the gathered posts were recorded. In addition, given causes and prevalences were extracted from package inserts (PIs), Summaries of product characteristics (SmPCs) and a scientific textbook. The collected data was matched to the corresponding daily defined doses (DDDs) in order to create a standardised database. Regarding the mentioned prevalences, a very heterogeneous distribution was found, depending on the social media platform used and the population size of the respective country. The results show that more drugs than currently known are linked to DIGO, and the prevalence rates described in various sources differ substantially from each other. Moreover, no linear relation between the size of the population and the percentage of contributing posts in the examined countries was found. Professionals should take different sources into consideration to decide which drug is likely to cause gingival overgrowth and which substance has a strong safety profile. Data from PIs and SmPCs alone are not sufficient to make reliable statements about ADRs and thus the drug safety of a particular active ingredient. Social media platforms offer a largely untapped opportunity for the early identification of adverse drug reactions and should be used for the post-market surveillance of pharmaceuticals.

**Supplementary Information:**

The online version contains supplementary material available at 10.1007/s00210-026-04983-w.

## Introduction

Gingival enlargement, previously described as “gingival hyperplasia” or “gingival hypertrophy” is a common adverse reaction caused by many different drugs. Anticonvulsants, calcium channel blockers and immunosuppressants are the groups with the highest risk of developing this undesirable effect (Brown and Arany 2015). But not only drugs can cause this condition, among others: systemic conditions, hereditary factors, allergies, local irritant factors, hormonal changes and vitamin deficiencies can lead to gingival overgrowth (Beaumont et al. 2017; Moffitt and Cohen 2013).

The underlying mechanisms on a molecular basis causing this condition are not yet fully understood. In general, the following cascade is used to explain the excessive overgrowth. Drugs blocking sodium and calcium channels lead to an insufficient ion influx into the cell which causes an intracellular deficiency of folic acid. This is followed by a disturbed activation of collagenases which in turn leads to reduced dismantling of connective tissue and an imbalance between synthesis and degradation of extracellular matrix (Droździk and Droździk 2023).

Due to the extensive swelling of the gums, patients suffer from unaesthetic appearance, insufficient dental hygiene aggravating the swelling, pain, bleeding and masticatory disfunction (Bharti and Bansal 2013). In addition, increasing inflammation of the gums leading to periodontitis culminating into systemic inflammation and potentially tooth loss are complications (Chojnacka‐Purpurowicz et al., 2022).

Possible therapeutic approaches contain the following: improvement of dental hygiene including scaling and root planing (SRP) as well as regular professional cleaning and modification or withdrawal of the causative medication if possible and reasonable. Surgical intervention and removal of the excessive tissue to create hygienic conditions are the last resort (Tungare and Paranjpe 2024). Unfortunately, drug-induced gingival overgrowth (DIGO) has a high recurrence rate which results in regular surgical tissue removal every 12–18 months (Zoheir and Hughes 2019). Further treatment options include the following: topical application of folic acid and systemic intake of antibacterial agents like azithromycin (Arya et al. 2011; Fuchs et al. 2019).

The best-known drugs to cause DIGO are phenytoin, nifedipine, ciclosporin and amlodipine (Brown and Arany 2015). Diltiazem, verapamil, valproic acid and carbamazepine are less familiar in this context. Reliable prevalence rates are hard to find because of the wide range that is reported from different sources describing one and the same substance (Droździk and Droździk 2023).

Due to these unfortunate circumstances, this study tries to depict as comprehensive as possible what drugs are most likely to cause DIGO, what prevalence can be expected and what possible benefit the monitoring of social media posts may have.

## Materials and methods

The following methodology was used for this paper: The English keywords “gingival hyperplasia”, “drug-induced gingival enlargement” and “drug-induced gingival overgrowth” as well as the German keyword “gingiva hyperplasie” were entered into the selected social media platforms (Facebook, YouTube, Instagram, TikTok and X), and the posts found were sorted according to origin, cause and treatment.

**Fig. 1:**
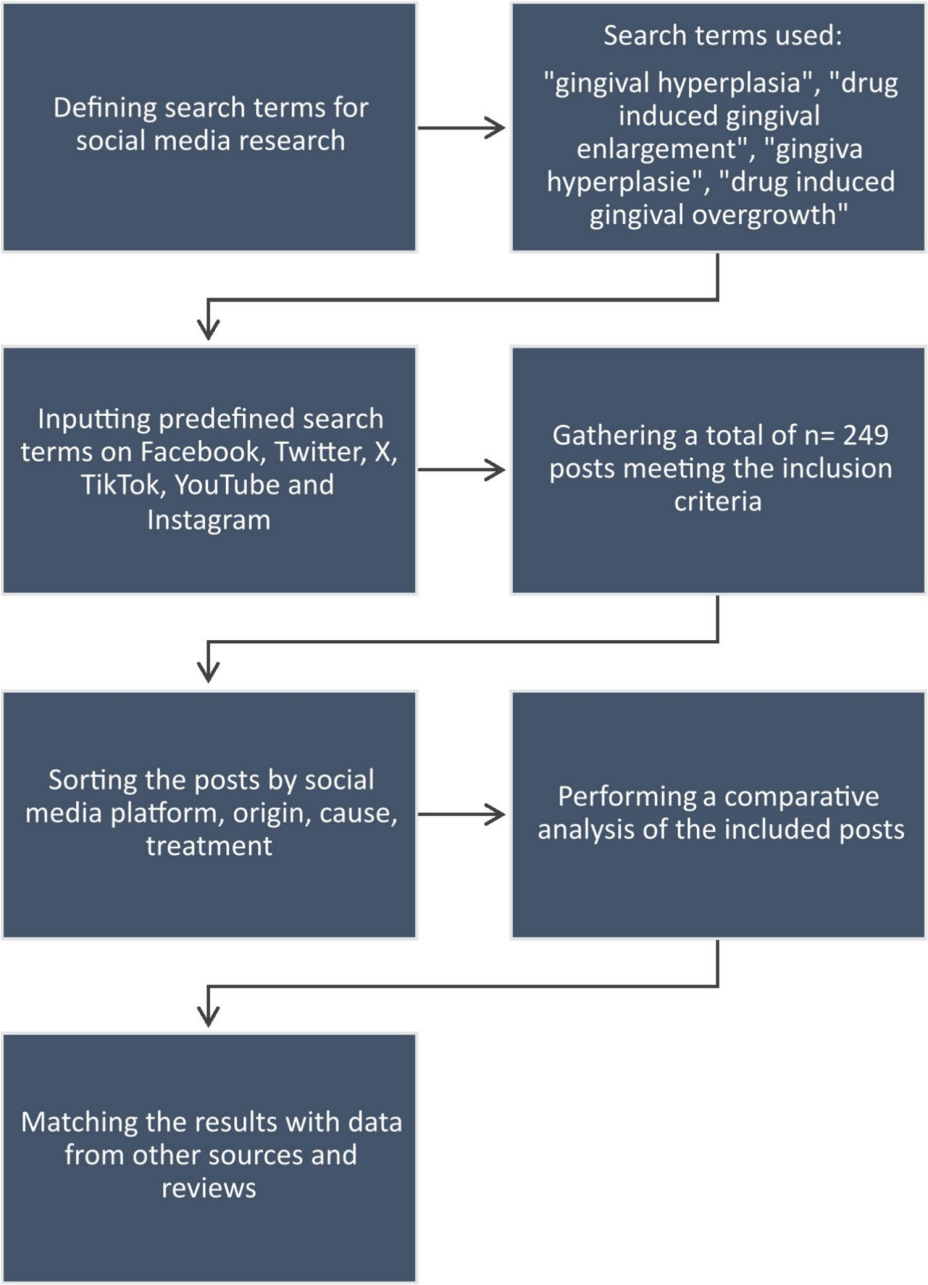
Presentation of the methods used for this paper on drug-induced gingival overgrowth

A comparative analysis was then carried out between the data collected from the summary of product characteristics, package inserts, social media posts and literature research. The data collected included the prevalence rates mentioned, possible risk factors, treatment options and the country of origin of the social media posts examined. The prevalences mentioned in the summary of product characteristics and package inserts for the ADR: “gingival overgrowth” and “gingival hyperplasia” were extracted and compared with the other sources. Varying severity and extent of DIGO were not taken into account, nor was the question of whether the diagnosis had been confirmed by a physician or was merely a symptom identified by the patient. The results obtained were compared with other sources and presented in comparative form (Fig. 1). The first author of the study was responsible for data evaluation.

### Comparative analysis

A comparative analysis of the described prevalence rates for drug-induced gingival overgrowth was performed including the following data sources:

1. Summary of product characteristics
2. Package inserts
3. Data from the social-media-research
4. Literature research, Scientific textbook (Goodman & Gilman’s 10.th edition “The Pharmacological Basis of Therapeutics”)

#### Comparing defined daily dose (DDD) and prevalence rates

To create a data set that is easier to compare, the prevalence rates given were set in relation to the prescription volume of the respective drug. The prevalence was calculated for 1 million DDD of the drug in question. To calculate this value, the prevalence (in %) was divided by the number of DDD (in millions).

#### Social-media-research

Social media posts from Facebook, YouTube, Instagram, TikTok and X (previously Twitter) were collected in February 2024. Inclusion and exclusion criteria were as follows:

All posts found using the above-mentioned search terms that contained data about the cause, treatment strategy or prevalence of DIGO were included in the analysis. Additionally, only posts in English or German were considered.

Furthermore, posts shared by the same account on multiple platforms were only registered once. Additionally, only posts created by humans were included; possible bot posts were not taken into account.

To achieve the largest possible database, there were no further explicit exclusion criteria.

To identify any bot accounts, we examined how many posts had been published by each profile and whether these were generic or individually designed. It was also checked how many followers the respective account had, whether additional information such as place of residence and employer was available, and whether the same post had been published repeatedly on different social media platforms. Only if these criteria potentially fitted a real person, it was assumed that it was not a bot account.

#### Ethical approval and data protection procedures

Data gathered in this study is publicly available via the mentioned social media platforms and was used for analysis. None of the posts included was located in “private areas” as in content for “friends” or “followers”. Accordingly, no individual informed consent was required for this analysis. Furthermore, no specific personal information was collected or published. It is therefore impossible to trace back the individual who published the analysed social media post, and complete anonymity of the users is ensured. In addition, the public benefit of recognising and assessing the prevalence of ADRs like DIGO outweighs the possible risks and harms of this study. Overall, the data examined is publicly available information that could be collected without interaction or intervention with the persons who posted the data. Consequently, no ethical approval was needed.

Subsequently all posts were categorised by the following:

1. Country of origin
2. Social media platform
3. Cause of gingival overgrowth
4. Treatment
5. Correctness

## Results and discussion

### Social-media-research:

**Fig. 2:**
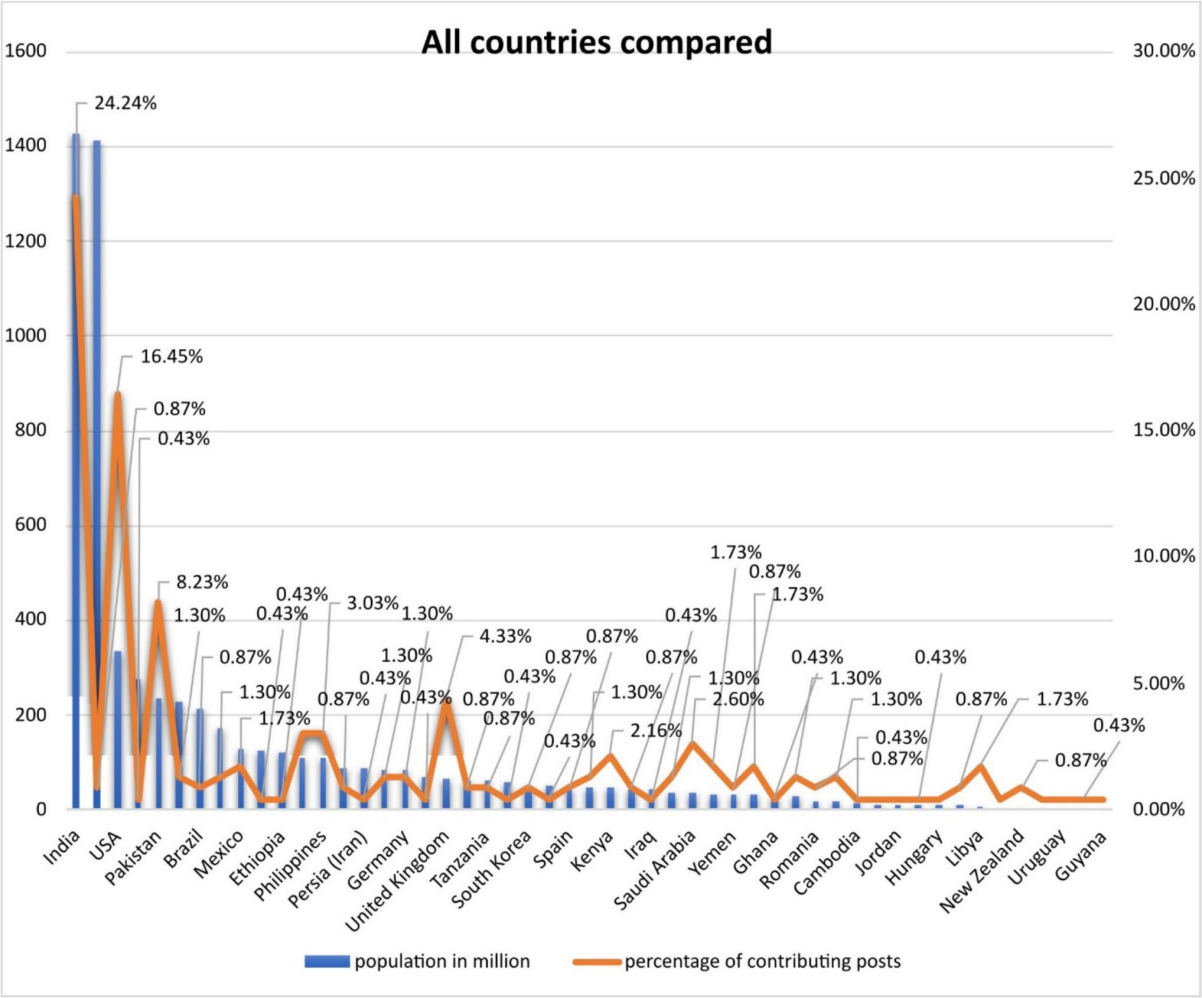
Chart showing the countries of origin of the analysed social media posts. The population figures for the individual countries are shown in comparison with the number of posts contributed as a percentage. The evaluation refers to a total of 231 posts whose country of origin could be determined

The social-media-research shows that there is no linear relation between the size of the population and the percentage of contributing posts in the examined countries (Fig. 2; Table 1). India, for example, with a population of ~ 1426 million contributed 24% of the collected posts. China in contrary with a population of ~ 1411 million contributed only 0.87% of the received posts. Regarding the size of the population, the countries contributed the following percentage of posts in descending order: USA (16.45%); Indonesia (0.43%); Pakistan (8.23%); Nigeria (1.3%); Brazil (0.87%); Bangladesh (1.3%); Mexico (1.73%) and Japan (0.43%). All other countries and their contributed posts are shown in the chart and table below.

**Table 1:** Tabular comparison of the individual countries in relation to their population and the percentage of posts from the respective country. Arranged in descending order starting with the country with the largest population. The corresponding sample size is the same as in Fig. 2 (231 posts)

All countries compared	Population in million	Percentage of contributing posts
India	1426	24.24%
China	1411	0.87%
USA	334	16.45%
Indonesia	275	0.43%
Pakistan	235	8.23%
Nigeria	229	1.30%
Brazil	214	0.87%
Bangladesh	172	1.30%
Mexico	127	1.73%
Japan	125	0.43%
Ethiopia	123	0.43%
Egypt	111	3.03%
Philippines	109	3.03%
Iran	87	0.87%
Persia (Iran)	87	0.43%
Turkey	85	1.30%
Germany	84	1.30%
Thailand	71	0.43%
UK	66	4.33%
South Africa	62	0.87%
Tanzania	61	0.87%
Italy	58	0.43%
South Korea	51	0.87%
Colombia	51	0.43%
Spain	48	0.87%
Uganda	47	1.30%
Kenya	47	2.16%
Algeria	45	0.87%
Iraq	43	0.43%
Canada	37	1.30%
Saudi Arabia	36	2.60%
Malaysia	33	1.73%
Yemen	33	0.87%
Peru	33	1.73%
Ghana	30	0.43%
Nepal	29	1.30%
Romania	19	0.87%
Ecuador	17	1.30%
Cambodia	16	0.43%
Kashmir	12	0.43%
Jordan	11	0.43%
Azerbaijan	10	0.43%
Hungary	9	0.43%
United Arab Emirates	9	0.87%
Libya	6	1.73%
Lebanon	5	0.43%
New Zealand	5	0.87%
Puerto Rico	3	0.43%
Uruguay	3	0.43%
Estonia	1	0.43%
Guyana	0.8	0.43%

Different factors can be used to explain these very mixed results between the examined countries (Table 1). On the one hand, an above-average incidence of DIGO in a population can lead to this specific medical problem receiving increased attention, which could be reflected in social media usage behaviour. On the other hand, it cannot be assumed that social media is used equally as a channel for the dissemination of medical knowledge or problems in all the countries considered here. In addition, there may not be comparable freedom of expression in all examined countries, and therefore, the censorship of posts on social media platforms obscures the actual frequency with which an adverse drug reaction such as DIGO occurs. The individual perception of gingival overgrowth and the individual suffering of each patient lead to a highly personal and therefore specific relevance of this topic, which is potentially reflected in social media usage behaviour. Because not every patient suffers equally from this ADR, not every patient has a high level of interest in finding solutions to their problem. It is therefore very difficult to accurately determine the absolute number of people affected based on the number of posts about DIGO. Nevertheless, conclusions about the relevance of this topic can be drawn from social media. A high number of posts tends to indicate that many people are affected, and that the observed ADR therefore represents a serious problem. In addition, it should be noted that the individual platforms are used to varying degrees in different countries, as the following charts illustrate.

### Social media platforms

**Fig. 3:**
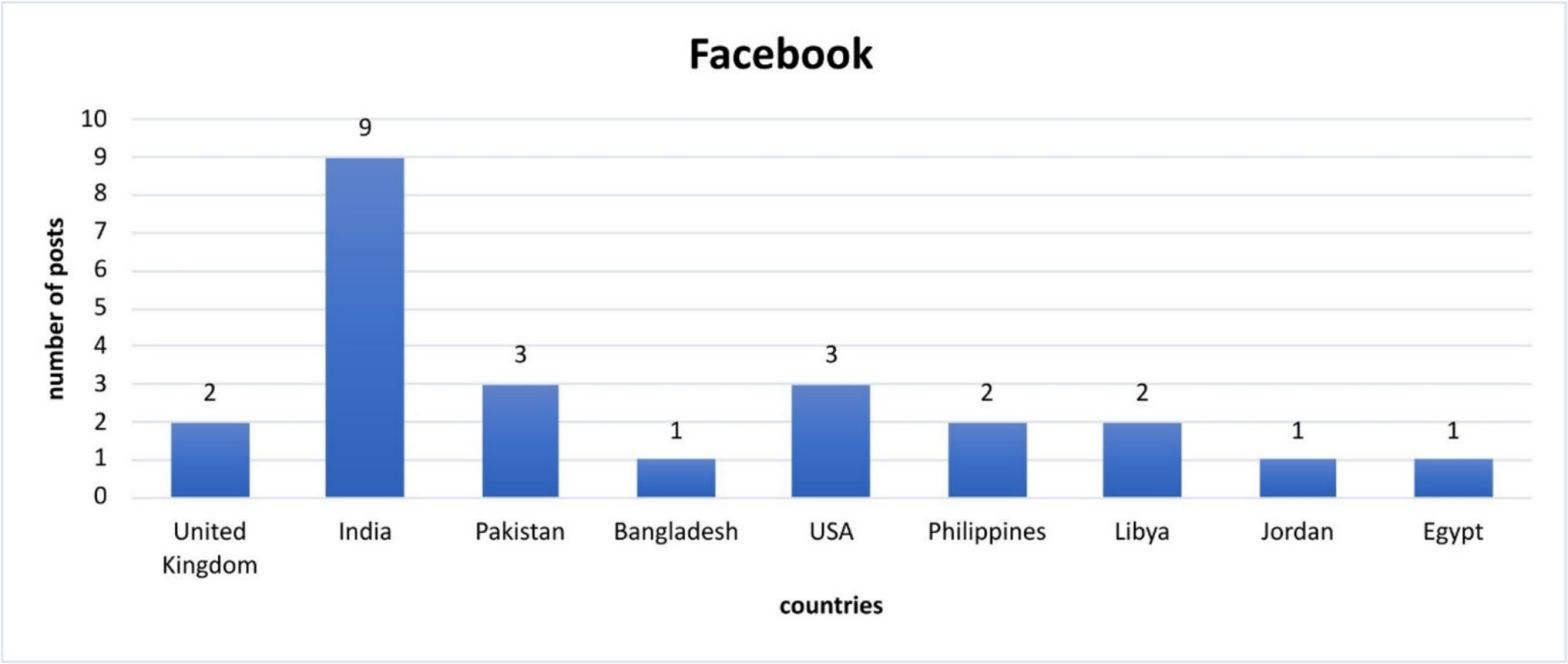
Evaluation and presentation of the Facebook posts and the respective countries of origin

In total, 24 Facebook posts were gathered and examined (Fig. 3). Out of these posts, two were from the UK, nine from India, three from Pakistan, one from Bangladesh, three from the USA, two from the Philippines, two from Libya, one from Jordan and one post from Egypt.

To avoid repetition, the gathered posts from YouTube, Instagram, TikTok and X are presented in the supplement. The evaluation follows the example above.

It is striking that most social media posts come from the USA, India and Pakistan. Only on TikTok was no post found from India, and only one post was found from Pakistan (Figs. 3–S4). This shows the differences in the relevance and use of various platforms in individual countries in an international comparison. It also shows the varying willingness of people of different nationalities and cultures to share medical information publicly on social media. As a result, it can be stated that there is a great deal of interest in medically relevant exchange via social media, particularly in the USA and India.

In India YouTube, X, Facebook and Instagram are mainly used to share information about DIGO, while TikTok does not seem to play a role. YouTube in particular has a lot of educational material and videos about the surgical treatment of DIGO. In contrast, all platforms are used in the USA, with the focus also on YouTube and X. TikTok, in particular, plays a crucial role in the dissemination of posts related to this ADR.

#### Causes of gingival overgrowth according to the social media posts

The following causes emerged from the examined social media posts. In descending order: 11.5% of the posts specified no cause, 10.7% stated phenytoin, 7% stated ciclosporin, 5.3% stated nifedipine, 5.2% stated poor oral hygiene/plaque/caries/tartar, 5.1% stated calcium channel blockers and 4% stated amlodipine. All other causes mentioned are listed in the table below.

Of the triggers for DIGO mentioned, it is particularly noticeable that phenytoin, ciclosporin and nifedipine are among the most frequently listed triggers. These are followed by amlodipine, verapamil and diltiazem. In a total of 14 posts analysed, valproic acid was mentioned as a trigger (Table 2). This is remarkable insofar as no link between valproic acid and DIGO could be established to date. The question therefore arises as to whether the relatively high number of posts may not be a warning sign that gingival overgrowth may occur with this drug in the context of an ADR. The same applies to ethosuximide, which was mentioned in 13 posts. In addition to specific drugs, numerous other factors are mentioned that are thought to lead to gingival overgrowth. These include particularly poor oral hygiene, systemic diseases and orthodontic treatment. Accordingly, an inflammatory reaction of the gingiva probably favours the development of gingival overgrowth.

**Table 2:** Overview of the causes of gingival overgrowth mentioned in the social media posts. All 249 posts were taken into account. The absolute number of causes mentioned as well as the percentage of the respective causes in comparison to all causes analysed are presented. Original search terms are given. In total, 882 potential causes were identified and taken into account

Cause	Absolute number	Percentage
Not specified	102	11.56%
Phenytoin	95	10.77%
Ciclosporin	62	7.03%
Nifedipine	47	5.33%
Poor oral hygiene/plaque/caries/tartar	46	5.22%
Calcium channel blockers	45	5.10%
Amlodipine	36	4.08%
Verapamil	33	3.74%
Anticonvulsants	32	3.63%
Diltiazem	27	3.06%
Systemic diseases	27	3.06%
Immunosuppressants	26	2.95%
**ADR**	26	2.95%
Orthodontic treatment	23	2.61%
Valproic acid	14	1.59%
Ethosuximide	13	1.47%
Inflammatory	13	1.47%
Felodipine	12	1.36%
Phenobarbital	12	1.36%
Neoplasia	12	1.36%
vigabatrin	10	1.13%
Apparent enlargement/false hyperplasia	10	1.13%
idiopathic	9	1.02%
Tacrolimus	9	1.02%
Lamotrigine	8	0.91%
Topiramate	8	0.91%
Ethotoin	8	0.91%
Mephenytoin	8	0.91%
Nicardipine	7	0.79%
Leukaemia	7	0.79%
Genetic	7	0.79%
Complete dentures	6	0.68%
Hormonal changes	6	0.68%
Primidone	5	0.57%
Nitrendipine	5	0.57%
Sirolimus	4	0.45%
Nimodipine	4	0.45%
Local factors (poor oral hygiene)/ defective restoration margins	4	0.45%
Erythromycin	4	0.45%
Isradipine	3	0.34%
Nisoldipine	3	0.34%
Methsuxinimide	3	0.34%
Succinimides	3	0.34%
Crown fracture/trauma	3	0.34%
Kaposi’s sarcoma	3	0.34%
Oxodipine	3	0.34%
Non inflammatory/other than local **factors/** **oro-dental cause**	3	0.34%
Carbamazepine	3	0.34%
Vitamin c deficiency	2	0.23%
Mouth breathing	2	0.23%
Herbal medicine	1	0.11%
Deeply inserting frenulum	1	0.11%
Without any identifiable cause	1	0.11%
Beta blockers	1	0.11%
Manidipine	1	0.11%
Hypertrichiosis	1	0.11%
Methotrexate	1	0.11%
Atropine	1	0.11%
Diphenoxylate/lomotil	1	0.11%
Eruptive	1	0.11%
Food impaction	1	0.11%
Tranexamic acid	1	0.11%
**NSAIDs**	1	0.11%
Oral contraceptives	1	0.11%
Dengue fever	1	0.11%
Clevidipine	1	0.11%
High blood pressure medication	1	0.11%
Corticosteroids	1	0.11%
Cyclophosphamide	1	0.11%

#### Pharmacological insights

Calcineurin inhibitors, anticonvulsants and calcium channel blockers all modulate Ca^2+^ and Na^+^ channels in the cell membrane (Ramírez-Rámiz et al. 2017). This modulation leads to a reduced influx of cations into the cells and thus to reduced folic acid uptake. This shared mechanism involving modulation of cellular ion channels leads to the development of DIGO and is the reason why these groups of active substances in particular lead to the aforementioned ADR. Furthermore, all three groups influence collagen turnover with a change in synthesis and collagenolysis, which increases fibrotic deposition in the extracellular matrix. Usually, inflammation, which is particularly prevalent in cases of poor oral hygiene, triggers a repair process in the affected tissue, which is likely to be disrupted by taking the above-mentioned drugs. On a cellular level, myofibroblasts can lead to tissue remodelling, as observed in DIGO, due to their profibrotic characteristics. Various growth factors (GF) and cytokines, which mediate the interaction between the extracellular matrix and cells, cause the imbalance between collagen synthesis and degradation that occurs in DIGO. Moreover, high concentrations of connective tissue GF (CTGF) and cytokines Interleukin-6 (IL-6) and Interleukin-1β (IL-1β) have been identified in DIGO. In general, the mechanism of development is very similar to healing and repair processes in the body.

Furthermore, it is assumed that the dysregulated cytokine concentration is primarily responsible for the development of DIGO and not the direct mechanism of action of the triggering medication (Trackman and Kantarci 2004). In contrast to other regions in the body and oral cavity, the interdental papilla is particularly susceptible to overgrowth due to its special composition of surface receptors, procollagen, fibronectin and growth factors (Ramírez-Rámiz et al. 2017).

#### Described treatment

The following treatment options were described in descending order: surgical procedures (34.4%), not specified (32.8%), improve oral hygiene/periodontal therapy (13.5%), medication change (9%), antibacterial mouthwash (2.2%) and antibacterial drugs (1.9%) (Table 3). Continuing with all other mentioned treatment options listed in the table below.

**Table 3:** Overview of the treatment options for gingival overgrowth mentioned in the examined social media posts. The analysed posts are shown in percentages and absolute numbers. In total, 311 possible treatment options were identified and taken into account

Treatment	Absolute number	Percentage
Surgical procedures	107	34.41%
Not specified	102	32.80%
Improve oral hygiene/ periodontal therapy	42	13.50%
Medication change	28	9.00%
Antibacterial mouthwash	7	2.25%
Antibacterial drugs/amoxicillin/ metronidazole	6	1.93%
Removal of the triggering cause	5	1.61%
Removal of local disruptive factors	3	0.96%
Protective splint	1	0.32%
Administration of folic acid (topical)	1	0.32%
Regular follow-up care	1	0.32%
Stopping bad habits	1	0.32%
Salt water rinse	1	0.32%
Azathioprine	1	0.32%
Steroids	1	0.32%
Administration of vitamins	1	0.32%
Prednisolone	1	0.32%
Cyclophosphamide	1	0.32%
Perboracep/oleozon	1	0.32%

Possible treatment options that emerged from the posts analysed: surgical methods for the treatment of DIGO were listed most frequently (34.4%). This was followed at some distance by improved oral hygiene and periodontal therapies (13.5%). A change in medication came in third place, described in 9% of the posts. In principle, these three treatment options are supported by current studies, although in a different order. Oral hygiene should be optimised first, followed by a change in medication, if possible. Only if these two treatment options have not brought the desired success, the excess tissue should be surgically removed. Antibacterial mouth rinses and antibacterial medication in general were also mentioned on social media later on. To date, there is no clear scientific evidence to support this theory. The following treatment options were mentioned so infrequently that they will not be further considered.

#### Correctness of the posts

All posts were examined and categorized in “incorrect” for scientifically disproven, “correct” for scientifically proven, “partially correct” for partly correct but not complete regarding the given information and “not verifiable” if no information was given that could be checked. Six percent of the posts were “incorrect”, 18.4% were “correct”, 64.3% were “partially correct” and 11.2% were “not verifiable”. Information given by the collected social media posts were compared to the current scientific literature. If current literature was found that refuted the statement in the social media post, the post was classified as “incorrect”. If the statement was supported by current scientific findings, the post was classified as “correct”. If the post examined contained only correct information about the possible cause of DIGO, but not about the treatment, or vice versa, it was classified as “partially correct”. If there was no information that could be verified, for example because only images or videos of existing gingival overgrowth were posted without mentioning a cause or treatment, the post in question was classified as “not verifiable”. Sample posts are attached in the supplement (Table S1).

**Fig. 4:**
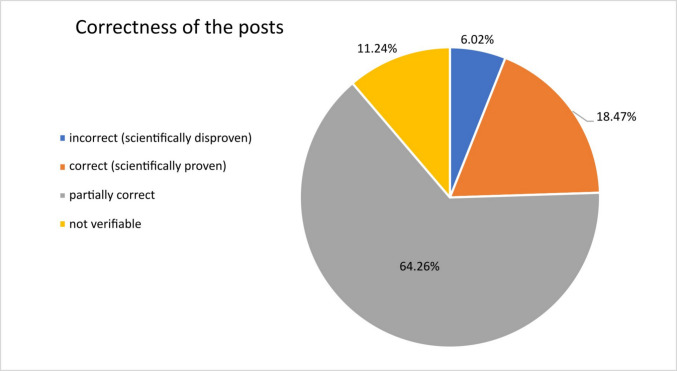
Presentation of the percentage distribution of all 249 analysed social media posts with regard to their accuracy

Overall, it can be observed that the majority of the posts analysed provide factually correct information, and only a small proportion is incorrect or scientifically refuted (Fig. 4). Many posts either only postulate the cause behind DIGO and do not suggest any treatment options, or only mention a possible treatment option without naming the underlying cause of the problem. Some posts only show pictures or videos of gingival overgrowth without going into more detail about causes or therapies. In such cases, the posts did not provide any clues that could have been verified. The second largest proportion of posts contained verifiable and scientifically proven information on the cause and treatment of DIGO. Conclusively, the high amount of correct and partially correct social media posts analysed leads to moderate optimism.

#### Methodological implications:

To set the following results into perspective and to make a comparison possible, a common data basis must be defined.

#### Data basis

- Social media research:882 registered causes = 100% causes were mentioned in the posts and evaluated
  - Of these, 102 were given as no cause or were unknown (11.56%)
- Package insert/SmPC: The frequencies given (very rare, rare, occasional, frequent, very frequent) were considered with the corresponding percentage values (< 0.01%, 0.01–0.1%, 0.1–1%, 1–10%, > 10%)
  -  < 0.01% was given as 0.01%
  - For 0.01–0.1%, the mean was calculated and given = 0.055%
  - For 0.1–1%, the mean was calculated and given = 0.55%
  - For 1–10%, the mean was calculated and given = 5.50%
  -  > 10% did not occur

#### Calcium channel blockers

**Fig. 5:**
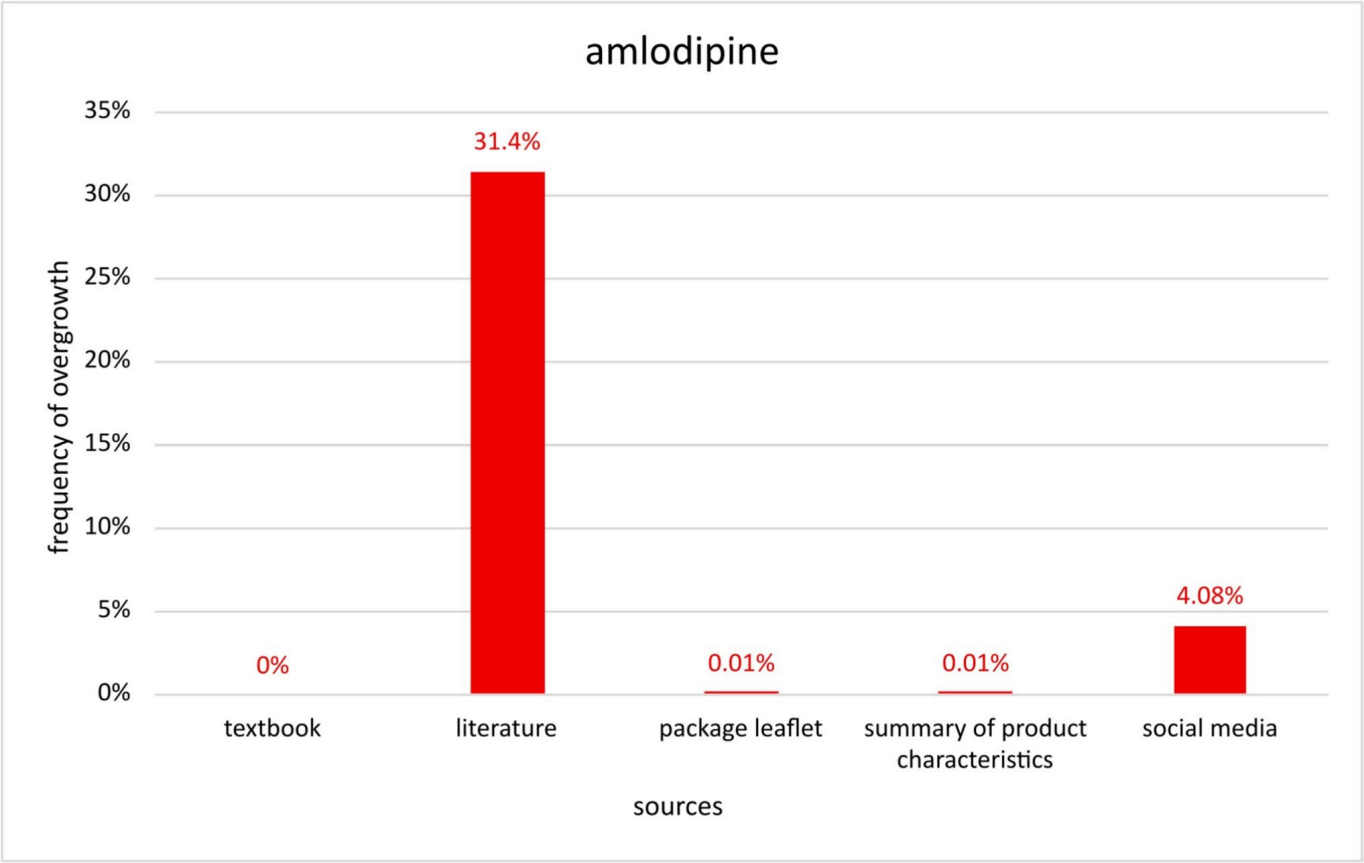
Presentation of the prevalence of gingival overgrowth induced by amlodipine. Comparison of the frequencies given in different sources

In order to determine the exact prevalence of amlodipine-induced gingival overgrowth, results from various sources were examined. Current literature found in “PubMed” suggests a prevalence of 31.4% whereas package leaflets and SmPC’s indicate a prevalence of 0.01%. The examined textbook does not mention amlodipine-induced gingival overgrowth whereas 4.08% were found from social media (Fig. 5). It certainly depends on the source and the underlying methods and data basis that lead to the calculated prevalence rate which is presented regarding DIGO, but the difference between 0.01 and 31% is far to big to be satisfied. Furthermore, it is remarkable that for all compared drugs, the results from PI’s and SmPC’s are by far the lowest given prevalence rates. Therefore, the question can be raised whether data from package inserts (PI) and SmPC’s are still reliable and accurate today?

#### Anticonvulsants

**Fig. 6:**
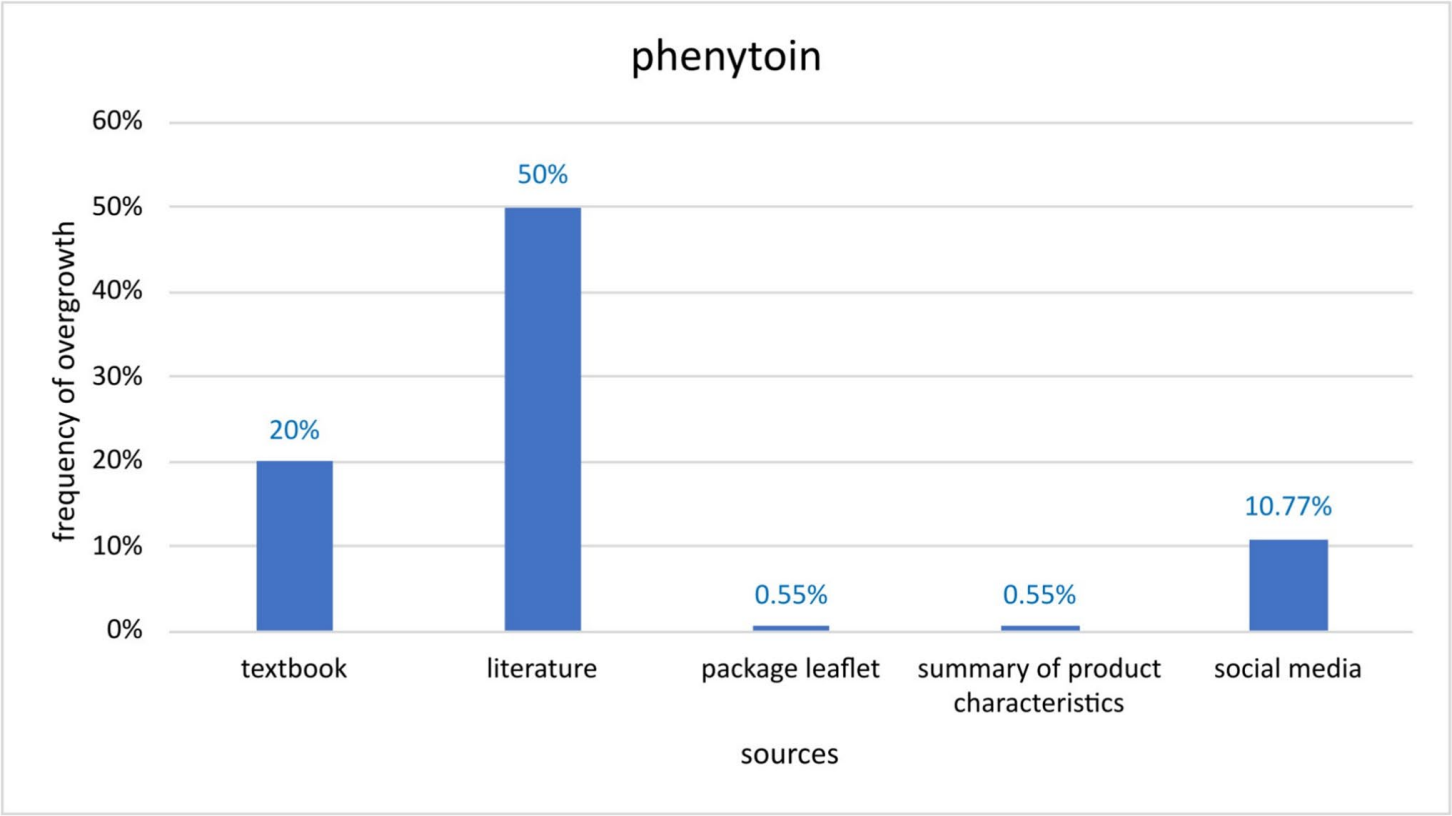
Presentation of the prevalence of gingival overgrowth induced by phenytoin. Comparison of the frequencies given in different sources

Regarding anticonvulsants, phenytoin is the drug that comes to mind. Comparing different sources, current literature presents the highest prevalence with 50% followed by the textbook with 20%, social media with 10.77% and PI’s plus SmPC’s state 0.55% respectively (Fig. 6). Similar tendencies can be shown between amlodipine- and phenytoin-induced GO. First of all, data from scientific literature shows the highest prevalence rates compared to PI’s and SmPC’s that show the lowest rates. Moreover, it stands out that for phenytoin, the scientific textbook and the current literature depict severely higher prevalences than PI’s and SmPC’s.

#### Immunosuppressants

**Fig. 7:**
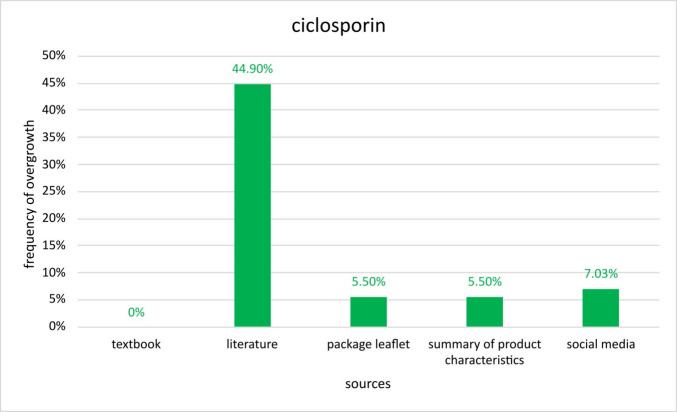
Presentation of the prevalence of gingival overgrowth induced by ciclosporin. Comparison of the frequencies given in different sources

The third important drug is ciclosporin belonging to the class of immunosuppressants. Once more, the collected data shows that the prevalence given in current literature is the highest with 44.9%. The textbook does not mention GO as an adverse effect of ciclosporin, whereas PI’s and SmPC’s claim that 5.5% is the correct rate. Data from social media suggests 7.03% (Fig. 7). It is noteworthy that the data outliers for ciclosporin in relation to the PI, SmPC and social media sources are not as high as for the other drugs. It seems like despite different databases, the results are closer together strengthening the significance of these numbers. Sorting the pharmaceuticals according to the highest mentioned prevalences, phenytoin ranks first, ciclosporin second, and amlodipine third. The consequence of this would be that patients taking phenytoin bear the greatest risk of developing GO followed by ciclosporin and amlodipine.

**Fig. 8:**
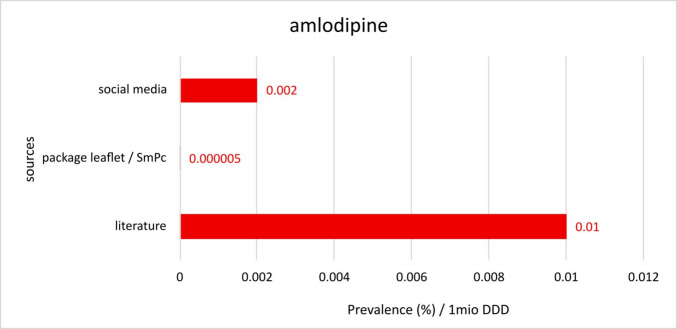
Comparison of the different prevalences per 1 million DDDs for amlodipine reported in the respective sources

**Fig. 9:**
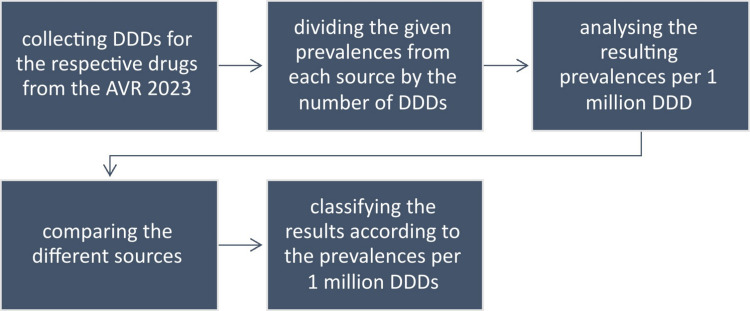
Flowchart visualising the data collection and analysis process for the prevalences per 1 million DDD

**Fig. 10:**
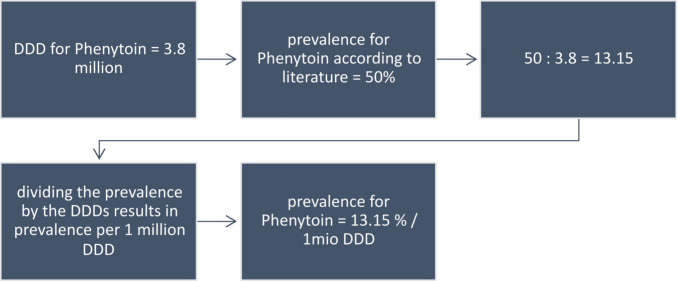
Demonstration of the calculation path for the prevalence per 1 million DDDs of phenytoin

**Fig. 11:**
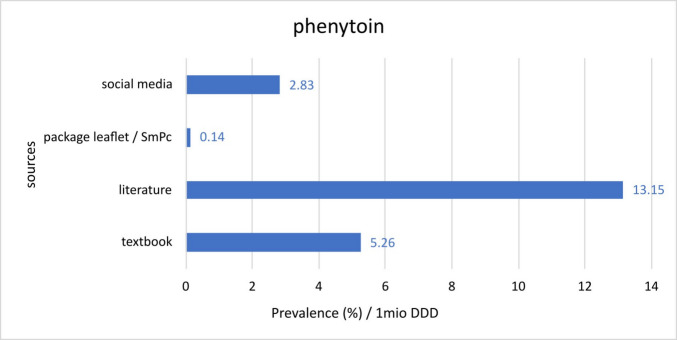
Comparison of the different prevalences per 1 million DDDs for phenytoin reported in the respective sources

**Fig. 12:**
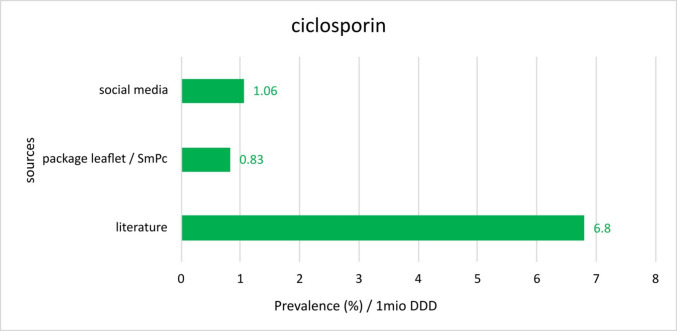
Comparison of different prevalences per 1 million DDDs for ciclosporin reported in the respective sources

To determine a reliable prevalence, the prescription rates of the affected drugs must be considered as well. Therefore, the “defined daily doses” (DDD) from the latest drug prescription report were used to calculate the corresponding prevalence for 1 million DDD’s. Information on the DDDs of the individual active substances was taken from the “Arzneiverordnungsreport 2023” (Ludwig et al. 2024). The data includes all drug prescriptions in Germany in 2023, registered by the drug index of the statutory health insurance “GKV-Arzneimittelindex” (GKV drug index). Therefore, the GKV drug index forms the basis for the annually published AVR, which was used as the source for the DDDs analysed here (Figs. 8, 9, 10, 11, and 12).

Considering the extremely high prescription rates of amlodipine, the following prevalences per 1 million DDD can be calculated: for social media 0.002, for PI’s and SmPC’s 0.000005 and for the literature 0.01 (Fig. 8). Especially with these results, it becomes clear how big the differences between the examined sources really are. The question arises how amlodipine-induced GO could be the most frequently observed drug-induced adverse reaction even though it has such a low prevalence.

Phenytoin on the other hand has much lower prescription rates compared to amlodipine which explains the higher numbers of prevalence per 1 million DDD. For the social media data, the prevalence rate is 2.83. PI’s and SmPC’s have the lowest rate at 0.14, the literature has a calculated rate of 13.15 and the textbook reports a prevalence of 5.26 (Fig. 11). Because of the higher prevalence of phenytoin inducing GO as an adverse reaction and the lower prescription rates, the overall prevalence rate per 1 million DDD’s is significantly higher compared to amlodipine and ciclosporin. This aspect must be taken into account when prescribing phenytoin or treating patients who regularly take this medicine.

Ciclosporin should not be underestimated as well. The immunosuppressant has the following prevalence rates: Social media reports 1.06, PI’s and SmPC’s 0.83 and literature 6.8 (Fig. 12). While the data from the social media research, the PI’s and SmPC’s, are relatively close together, prevalence rates from the literature are significantly higher. Similar to phenytoin, higher prevalence rates and a lower number of prescriptions for ciclosporin also result in higher prevalence rates per 1 million DDD’s. One possible explanation for the high figures from the literature could be small observation groups or a small number of studies leading to a more frequent observation of adverse drug reactions.

**Fig. 13:**
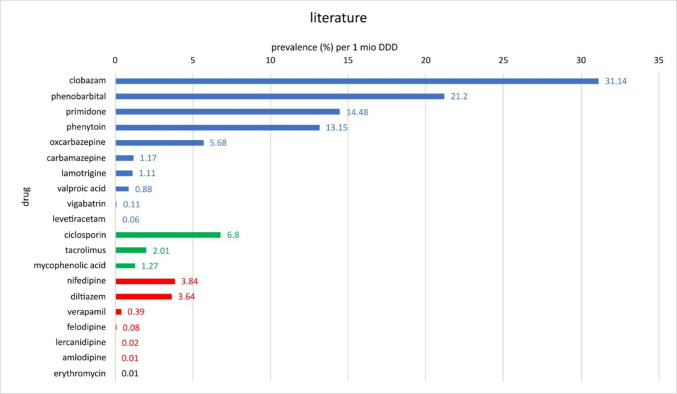
Overview of the various drugs with the associated prevalences per 1 million DDDs with data from the literature. Blue-coloured bars indicate that the drugs belong to anticonvulsants; green-coloured bars indicate immunosuppressants; red-coloured bars indicate calcium channel blockers; and black-coloured bars indicate different groups of pharmaceuticals

**Fig. 14:**
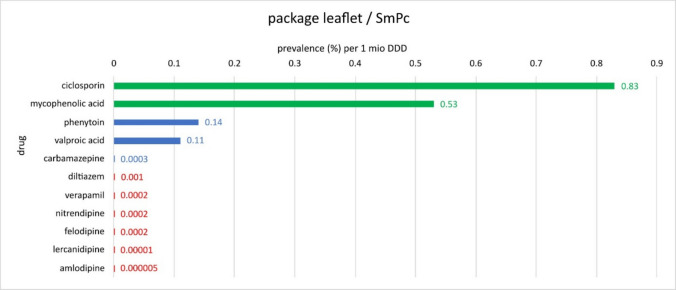
Overview of the various drugs with the associated prevalences per 1 million DDDs with data from the analysed package inserts and SmPCs. Blue bars indicate that the drugs belong to anticonvulsants; green bars indicate immunosuppressants; and red bars indicate calcium channel blockers

**Fig. 15:**
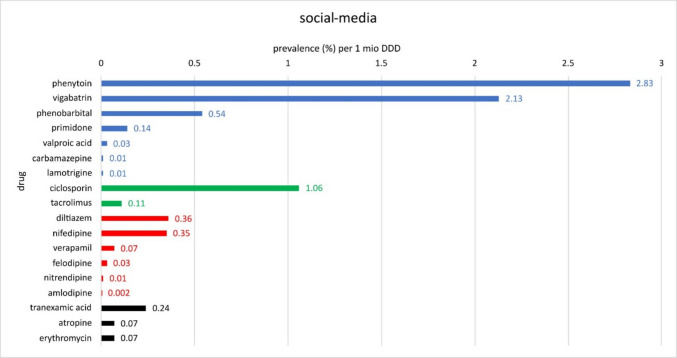
Overview of the various drugs with the associated prevalences per 1 million DDDs with data from the 249 analysed social media post. Blue coloured bars indicate that the drugs belong to anticonvulsants, green indicates immunosuppressants, red calcium channel blockers and black different groups of pharmaceuticals

The colour coding in Figs. 13, 14, and 15 divides the active substances into the following: anticonvulsants (blue), immunosuppressants (green), calcium channel blockers (red) and other groups of active substances (black).

A comparison of the data for the various active substances from one source each reveals the following distribution: Following the data acquired from the literature, clobazam has the highest prevalence per 1 million DDD (31.1). This is followed at some distance by phenobarbital (21.2), primidone (14.48), phenytoin (13.15) and ciclosporin (6.8), followed by oxcarbazepine (5.68), nifedipine (3.84), diltiazem (3.64), tacrolimus (2.01), mycophenolic acid (1.27), carbamazepine (1.17), lamotrigine (1.11), valproic acid (0.88), verapamil (0.39), vigabatrin (0.11), felodipine (0.08), levetiracetam (0.06), lercanidipine (0.02), erythromycin (0.01) and, in last place, amlodipine (0.01) (Fig. 13). The low prevalence of calcium channel blockers is particularly striking, with amlodipine in last place. Anticonvulsants, on the other hand, have by far the highest probability of triggering drug-induced gingival overgrowth. Looking at the data independently of the DDD, amlodipine is one of the drugs with the highest prevalence of inducing gingival overgrowth. It can therefore be concluded that the enormously high number of prescriptions for amlodipine leads to a relativisation of the high prevalence of this active substance (Fig. 13).

Data from the package leaflets and SmPCs show the following order: Ciclosporin (0.83) has the highest prevalence per 1 million DDD, followed by mycophenolic acid (0.53), phenytoin (0.14) and valproic acid (0.11), diltiazem (0.001), carbamazepine (0.0003), verapamil (0.0002), nitrendipine (0.0002), felodipine (0.0002), lercanidipine (0.00001) and finally amlodipine with (0.000005) (Fig. 14). Here, too, it is noticeable that amlodipine has by far the lowest prevalence of all the drugs listed. According to PIs and SmPCs, the immunosuppressant ciclosporin is the frontrunner, followed by various anticonvulsants. Accordingly, the data from the literature and those just mentioned are consistent in that anticonvulsants are among the agents with the highest prevalence of DIGO (Fig. 14).

Data from the social media analysis also show the highest prevalence per 1 million DDDs for phenytoin (2.83) (Fig. 15). This is followed by vigabatrin (2.13), ciclosporin (1.06), phenobarbital (0.54), diltiazem (0.36) and nifedipine (0.35). This is followed by tranexamic acid (0.24), primidone (0.14), tacrolimus (0.11), atropine (0.07), erythromycin (0.07), verapamil (0.07), felodipine (0.03) and valproic acid (0.03). Lastly, carbamazepine with 0.01% per 1 million DDDs, lamotrigine and nitrendipine also with 0.01 and amlodipine with 0.002 (Fig. 15).

**Fig. 16:**
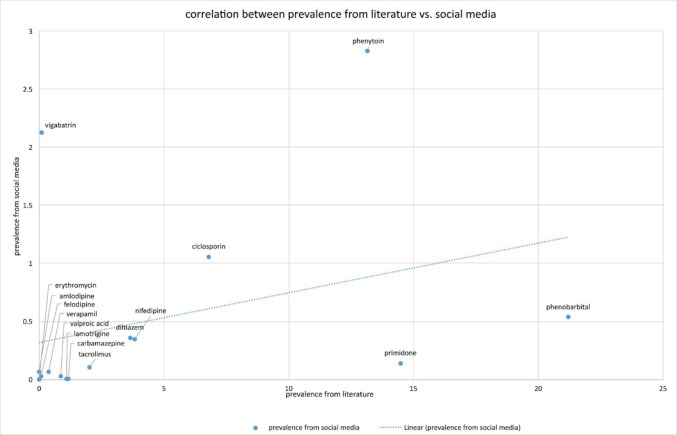
Illustration of the correlation between the data from scientific literature and social media. The prevalences (%) per 1 million DDDs were used

A correlation analysis of the data from scientific literature and social media shows that there is no linear correlation (Fig. 16). The surveyed prevalences (%) per 1 million DDDs of the respective drugs were compared with each other. Among others, the following drugs were analysed: amlodipine, felodipine, valproic acid, lamotrigine, carbamazepine, ciclosporin, phenytoin and phenobarbital. A weak positive correlation with *r* = 0.326 was found, but this was not statistically significant (*p* = 0.235). These results show that there is no significant correlation between the prevalence rates recorded in literature and social media. It also shows that the collection of reliable prevalences is extremely difficult, even with the help of different sources, with each source providing different data.

From the data presented, the question arises as to why the package leaflets and SmPC’s consistently show the lowest prevalence rates compared to the other sources. One conceivable explanation for this would be that the randomised controlled trials used as a basis for the observation of adverse drug reactions are too small to correctly depict all adverse drug reactions and their frequencies. Moreover, many RCTs do not have the statistical power to reliably detect common ADRs. (Wahab et al. 2013). The problem especially with rare ADRs is that very large numbers of participants and long observation periods are necessary to identify them reliably prior to market approval (Berlin et al. 2008; Lessing et al. 2010). Particularly in the case of newly approved active substances intended for long-term therapy, only approximately 80% of approved drugs meet the minimum requirements prior to market launch (Duijnhoven et al. 2013).

The minimum requirements for studies that should be conducted prior to market authorisation to identify common ADRs are defined in the “ICH E1 Guideline” (The Extent of Population Exposure to Assess Clinical Safety for Drugs Intended for Long-Term Treatment of Non-Life-Threatening Conditions). This guideline was published by the International Council for Harmonisation (ICH) and is intended to support the harmonisation of international drug development. Among other requirements, it specifies that at least 300 patients should be treated with the drug in question for at least 6 months to obtain initial information on long-term safety. In addition, at least 100 patients should be treated for at least 1 year to assess prolonged safety. At least 1500 patients should be exposed to the active substance in short- and long-term to obtain a sufficient safety profile. Furthermore, the patient population should reflect the target population in terms of age, comorbidities and co-medication. Finally, exposure should reflect the intended clinical use in terms of dosage, duration and indication as closely as possible (European Medicines Agency, 1995).

Overall, the risks associated with new drugs can only be assessed to a limited extent prior to market authorisation and that post-marketing surveillance and pharmacovigilance are essential in order to establish a complete and reliable safety profile.

Another problem with package leaflets and SmPCs is that no regular updates of the available data are carried out to include or update new findings regarding adverse drug reactions and other essential information in the package leaflets. This inevitably leads to misinformation, treatment errors and lack of knowledge on the part of both doctors and patients (Drelich et al. 2022).

Studies that are carried out before a drug is approved and are sponsored by the industry almost always lead to a favourable result for the sponsors. This could lead to relevant problems such as adverse drug reactions being checked before the start of the study and the study design subsequently being adapted so that as few problems as possible occur in order to bring the drug to market quickly. As a result, certain adverse drug reactions may not be detected at all or only very rarely, and the package leaflets may be written on the basis of this data (Fries and Krishnan 2004).

To identify adverse drug reactions after the introduction of a drug, it is essential to use a spontaneous reporting system. This enables continuous monitoring of drug safety and the risk-adapted use of these drugs. However, only by taking this data into account and regularly adapting the publicly available information, it is possible to determine which risks and adverse drug reactions a drug actually contains (Noguchi et al. 2021).

#### Role of social media in pharmacovigilance

Social media posts have the ability to reflect the overall opinion of a certain group among our society (Castillo et al. 2022). Thus, there is the potential of using social media for scientific research. For example, current studies try to prove that social media posts have a significant value for spontaneous reporting system databases. These posts should be used as an early detection system to improve the current databases and detect adverse drug reactions even earlier than today (Powell et al. 2016). The analysis of adverse drug reactions in social media is a relatively new but promising method in science with the first publications in 2012 (Yang et al. 2012). In contrast to spontaneous reporting systems, one of the advantages of social media is that there is significantly less “underreporting”, especially in case of unknown adverse drug reactions, which is often problematic with conventional post-marketing surveillance methods (Banerjee and Ingate 2012; Yang et al. 2012).

Mild, non-serious adverse drug reactions in particular could be recognised earlier and more reliably through social media than through conventional pharmacovigilance systems (Golder et al. 2024). However, despite the high sensitivity that social media brings with it, the results that were statistically analysed are rather imprecise (Caster et al. 2018). The frequencies analysed were generally lower for social media (Golder et al. 2024). Nevertheless, social media also offers the opportunity to provide additional data on adverse drug reactions via automated processes (Li et al. 2025).

Cultural differences in social media usage patterns must not be overlooked. It has been shown that developing countries in particular are among the nations that use social media the most (Ittefaq et al. 2022). Nevertheless, only those who can afford an internet-enabled mobile device and are able to use it can participate in the increasing communication between public healthcare providers such as the World Health Organization (WHO) or local health facilities. This means that very poor people, children and elderly people may be cut off from access to health-related information. However, it is precisely these population groups that are often particularly vulnerable and affected by health problems. In addition, different usage patterns can be observed with regard to the different platforms. It was shown that WhatsApp is the most commonly used platform in Pakistan, followed by YouTube and Facebook, with Twitter being the least commonly used (Ittefaq et al. 2022). Significant differences were also found in relation to gender. Only a fraction of Pakistani women use Twitter or Facebook compared to men (Ittefaq et al. 2022).

Age also has a significant influence on social media usage behaviour. In the USA, the majority of the population who use the internet are between 18 and 24 years old. With increasing age, the proportion of internet users drops sharply (Chou et al. 2009). This age distribution must be taken into account when communicating health-related information.

Another danger lies in the censorship of information on social media platforms. Although all major platforms are committed to freedom of speech, there are repeated instances of disinformation campaigns and censorship of posts that do not fit into the generally accepted narrative. Politically influenced moderation of posts therefore leads to the manipulation of information and opinion (Stjernfelt and Lauritzen 2020). An example of the censorship of posts was seen during the COVID-19 pandemic. Platforms such as Facebook, Twitter and YouTube wanted to stop misinformation about the virus and highlight information from public authorities such as the WHO (Niemiec 2020). Even though the focused sharing of information by public health authorities is certainly a desirable goal, it must not be allowed to suffocate scientific discussion about the advantages and disadvantages of various measures. Scientific discourse on health-related topics must be possible on social media without government institutions or a few private companies deciding which posts are published and which are deleted.

In this study, 249 posts from five of the biggest social media platforms worldwide were examined to search for new or rare triggers of gingival overgrowth. Besides the commonly known triggers of gingival overgrowth like drugs, systemic diseases, neoplasia and false enlargement (Beaumont et al. 2017; Moffitt and Cohen 2013), some social media posts suggest other reasons behind this undesirable effect. More precisely further drugs than the well-known calcium channel blockers, anticonvulsants and immunosuppressants are mentioned in the examined posts. These possible triggers must be examined closely, and a scientifically based decision must be made, whether the mentioned trigger really leads to gingival overgrowth or not. The most frequently named triggers are the following: phenytoin, ciclosporin, nifedipine, poor oral hygiene, calcium channel blockers, amlodipine, verapamil, anticonvulsants, diltiazem and systemic diseases (Table 2). Although nifedipine, amlodipine, verapamil and diltiazem belong to the group of calcium channel blockers, the drugs are far more often mentioned as the reason behind drug-induced gingival overgrowth than the publicly known prevalence rates suggest. These exceptionally high rates of posts about these specific drugs could imply that the currently known prevalence rates are lower than the adverse reactions actually occur in reality. Another possible explanation could be that the prescription rates of these certain drugs are so high that the number of posts actually correlate to the specified prevalence rates. To verify this thesis, the prevalence in relation to the “defined daily dose” (DDD) was calculated, and the results for 1 million DDD respectively for each drug were presented graphically (Figs. 8, 11, and 12). Some of the less often named triggers which are nevertheless worth mentioning are ther following: valproic acid, ethosuximide, felodipine, phenobarbital, false hyperplasia, tacrolimus, leukaemia, genetic, hormonal changes and primidone (Table 2).

Alongside to the posted triggers, the preferred treatment options were also named on social media. In general, the following treatment regimen for gingival overgrowth is widely accepted: First of all, the patient’s oral hygiene should be improved, and periodontal treatment should be carried out if necessary. Secondly if no improvement of the condition is achieved, dose adjustment or changing of the triggering drugs should be done if possible. Last of all, surgical removal of the extensive tissue and recreation of hygienic conditions should be carried out to improve the patient’s condition (Tungare and Paranjpe 2024). The most frequently mentioned treatment option was “surgical procedures” in 34% of the posts, followed by “improvement of oral hygiene” in 13.5% and “medication change” in 9% (Table 3). These results are consistent with the above-mentioned preferred treatment regimen.

#### Review of reviews on DIGO

An overview of current reviews on the topic of “DIGO” illustrates how heterogeneous the data particularly regarding the drug-specific prevalence is (Table 4). It should not be forgotten that different methods and different underlying data lead to different prevalences. From an overall view of the data, it is important to recognise a trend or a range in which the actual frequency with which the respective drug triggers gingival overgrowth is found.

**Table 4:** Overview of the analysed reviews and the prevalence of DIGO mentioned therein. Presentation of the respective prevalence for the associated drug

Review	Amlodipine	Nifedipine	Diltiazem	Verapamil	Ciclosporin	Tacrolimus	Phenytoin	Lamotrigine	Carbamazepine	Phenobarbital	Valproic acid
Chojnacka‐Purpurowicz, et al. (2022)					8–85%						
Droździk, and Droździk, (2023)	1.7–94.8%	6.3–83%	2.2–20%	19%	22.4–44.9%		20–65.4%	61%	15.1–32%	19.7–53%	17.5–44%
Zhang et al. (2022)	6.81%	17.93%	3.85%	3.70%	44.89%	4.59%	18.22%				
Bajkovec et al. (2021)	10–20%	15–85%	10–20%	10–20%	8.3–67%	14%					
Brown, and Arany, (2015)	30–50%	30–50%	30–50%	30–50%	7–80%		10–83%				
Sharma et al. (2020)							Nearly 85%, clinically significant approximately 50%				
Bhombe (2020)	6–15%	6–15%	5–20%	< 5%	adults: 25–30%, children > 70%		50%	50%	50%	50%	50%

#### Clinical implications for dentists and prescribers

The social media research shows that there is no linear relation between the size of the population and the percentage of contributing posts in the examined countries. This means that regardless of the population size of a specific country, an above-average number of DIGO cases may occur, and dentists must be vigilant in identifying the underlying triggers. Furthermore, social media usage behaviour shows that there is a common interest in medical information on social media platforms. Accordingly, informative posts by dentists and physicians could lead to better education of those affected and thus to an increased treatment rate, as low-threshold treatment approaches are presented.

Based on the triggers of gingival overgrowth collected on social media platforms, several clinically relevant aspects emerge that may be overlooked in everyday practice or are not known to all practitioners. For example, previously unknown triggers such as valproic acid, ethosuximide or vigabatrin are mentioned in connection with DIGO. However, rare non-pharmaceutical triggers should also be kept in mind. Various systemic diseases such as leukaemia, vitamin deficiency, neoplasms and hormone fluctuations are relevant triggers that clinicians should be aware of. Overall, it is therefore advisable to attend regular seminars on various aspects of oral health in order to stay up to date with the latest scientific research.

A similar situation can be observed with regard to the treatment options mentioned. Surgical procedures are the most common choice for treating DIGO on social media platforms. However, practitioners should be aware that a defined treatment cascade is scientifically recommended. As mentioned above, treatment of DIGO should begin with optimisation of oral hygiene and, if possible, a change in the patient’s medication. If these therapeutic approaches are not possible, insufficient or do not lead to a satisfactory improvement of the overgrowth quickly enough, surgical removal of the excessive tissue should be performed. Stable conditions on a long-term basis can only be achieved by combining these therapeutic methods. Furthermore, regular checks should be carried out to see whether new, improved and scientifically validated therapeutic concepts have been developed.

To assess the risk of a drug triggering certain ADRs, it is essential to know the most accurate prevalences possible. Only by comparing different active ingredients and their associated prevalences, the most suitable drug for the patient can be found. However, the frequencies investigated, for example for amlodipine, vary between 0.01 and 31.4% depending on the selected source (Fig. 5). Due to this variation, it is very difficult to rely on a single source and use the information provided to estimate how high the patient’s risk of developing DIGO actually is. As a result, it is practically impossible to minimise the risk accordingly.

With regard to DIGO, it is particularly concerning that there is no consistent information available on how frequently certain drugs can lead to this ADR. Such an extremely heterogeneous database makes it considerably more difficult for physicians to create a reliable risk profile and make a rational decision regarding the selection of the best drug. Based on the results presented above, it is advisable to not only use information from PIs and SmPCs for assessment, but also to take a look at the current literature and social media. There is a risk that PIs and SmPCs are not up to date, as they may not have been revised or updated for several years. Within the European Union (EU), regular checks and revisions of PIs and SmPCs are required by law, but the time periods within which the marketing authorisation holders must carry them out are not specified. To obtain the most up-to-date overview of the risk profile of various active substances, dentists and physicians should therefore consult scientific literature and then select the appropriate active substance.

To support the suspicion that valproic acid, ethosuximide and lamotrigine can also lead to DIGO, current scientific research on this topic was examined. Gallo et al., (2021) report prevalences of 44% for valproic acid, 61% for lamotrigine and 71% for oxcarbazepine. Rajendran, (2022) also describes a case of lamotrigine-induced gingival overgrowth. In addition, Anderson et al., (1997) describe a case of severe gingival overgrowth while taking valproic acid. Further evidence for this ADR with valproic acid and sodium valproate is provided by Bondon-Guitton et al., (2012); Tan et al., (2004); Syrjänen and Syrjänen, (1979); Behari, (1991); and Patil et al., (2014). Moreover, cases of gingival overgrowth have also been described in patients taking ethosuximide (Singh et al. 2021; Karn et al. 2022; Cláudio et al. 2020).

### Limitations

The data collected and analysed correspond to a sample and may not be all-encompassing or complete. Social media posts harbour a cluster risk due to “group of friends” and algorithms. It is very difficult to check for accuracy, as many posts are incomplete or ambiguous, and some theses have not yet been verified in the current study situation. Only one person (the first author) collected and coded data. It will be interesting to see whether data collection and data coding by multiple investigators yield different results. Finally, the stated frequencies for the occurrence of gingival overgrowth are only comparable to a limited extent, as the basic data bases differ significantly depending on the source.

## Conclusions

In conclusion, it is fair to say that social media can be a valuable addition in the recording of rare adverse drug reactions. Those affected individuals report their symptoms and are on the lookout for possible therapies. All in all, the posts are largely correct, even if there is of course a certain risk of spreading false facts. Serious differences that exist within the sources considered with regard to the causes of DIGO should be examined in detail in order to be able to assess all risk factors as far as possible. Especially, drugs with a particularly high number of prescriptions should be subject to regular surveillance.

Nevertheless, caution is required when interpreting the prevalence data, as different sources produce very different results depending on the underlying database. In addition, the current prescription rates should be considered in order to see the prevalence in relation to the actual quantity of the drug dispensed.

### Further perspectives

For future research, there is a growing list of drugs that are suspected of causing gum disease including gingival overgrowth, but there is no detailed review or confirmation of this link yet. In addition, the exact mechanisms of drug-induced gingival overgrowth have still not been conclusively clarified, so further studies are necessary to understand these complex relationships.

## Supplementary Information

Below is the link to the electronic supplementary material.

ESM 1 (490 KB DOCX)

## Data Availability

All source data for this study are available upon reasonable request from the authors.

## Abbreviations

ADR: Adverse drug reaction
AVR: Arzneiverordnungsreport (Drug prescription report)
CCB: Calcium channel blocker
DDD: Defined daily dose
DIGO: Drug-induced gingival overgrowth
GF: Growth factor
GO: Gingival overgrowth
PI: Package inserts/package leaflets
RCT: Randomised controlled trial
SmPC: Summary of product characteristics
SRP: Scaling and root planing
