# Supplementary material for: Social media data and its potential for pharmacovigilance: a comparative analysis of reported prevalences regarding drug-induced gingival overgrowth (DIGO)
Source: Naunyn Schmiedebergs Arch Pharmacol. 2026 Jan 23;399(7):9673–94. doi: 10.1007/s00210-026-04983-w (PMC13152936; doi:10.1007/s00210-026-04983-w)
Supplement: Supplementary file 1 — (490 KB DOCX) [file 210_2026_4983_MOESM1_ESM.docx]

# **Supplemental material:**

# **Social media data and its potential for pharmacovigilance**

# **A comparative analysis of reported prevalences regarding drug induced gingival overgrowth (DIGO)**

**Philipp Friedrich Georg Ried**

**and**

**Roland Seifert**

**Institute of Pharmacology
Hannover Medical School
D-30625 Hannover, Germany
Corresponding author: Roland Seifert
seifert.roland@mh-hannover.de**

***Table S1*** Presentation of exemplary social media posts and their classification into the categories “correct”, “incorrect”, “partially correct” and “not verifiable”.

| Correct | Incorrect | Partially correct | Not verifiable |
| --- | --- | --- | --- |
| Phenytoin is a possible cause of DIGO. It is therefore important to improve dental hygiene and to get surgical intervention in severe cases. | Only nifedipine leads to gingival overgrowth, other calcium channel blockers don’t. | Poor oral hygiene combined with various drugs like amlodipine or phenytoin can cause DIGO | Before and after pictures of gingival overgrowth |
| Anticonvulsants, immunosuppressants and calcium channel blockers are likely to cause DIGO. Periodontal treatment and gingivectomy are suitable treatment options. | Gingival overgrowth doesn’t occur in edentulous regions. | Changing the causative medication and surgical removal may be successful treatment options. | Gingival overgrowth can occur in various patients. |


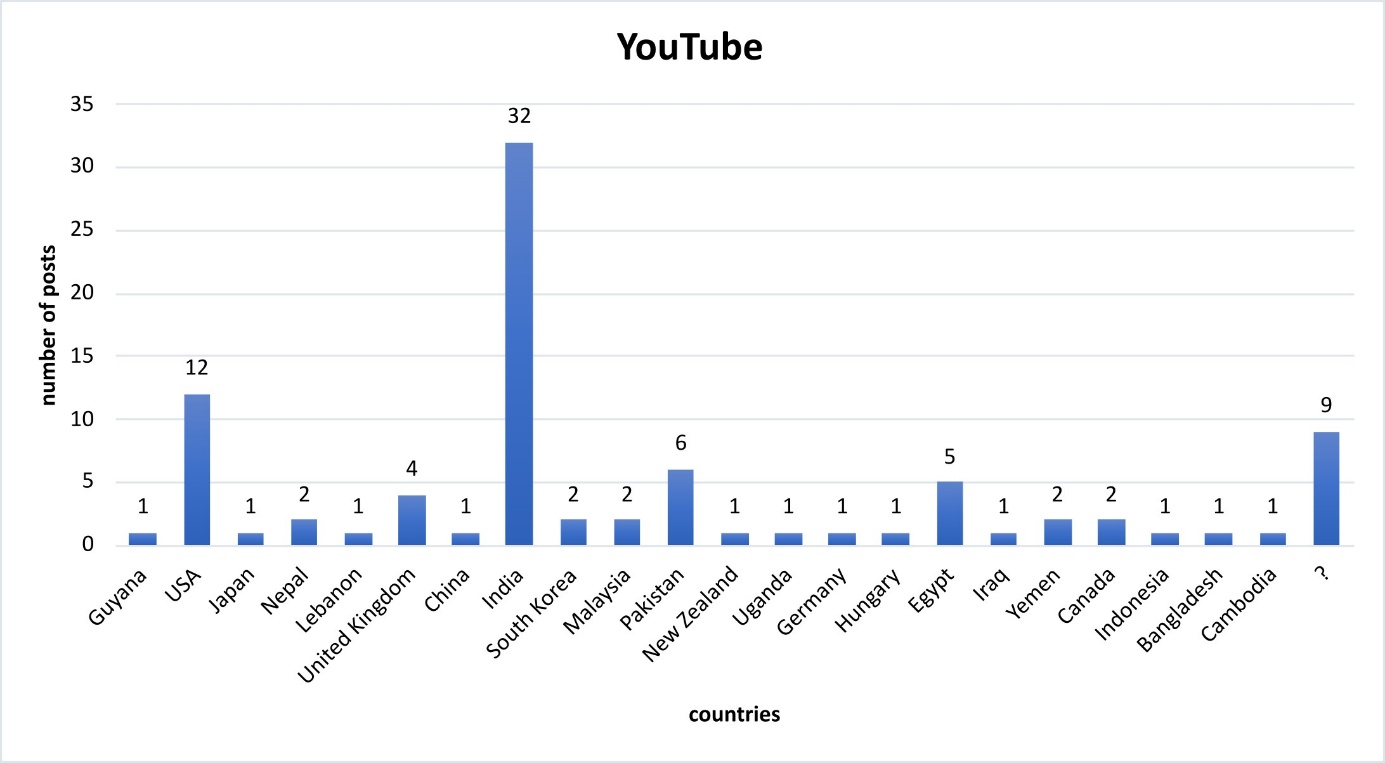
 ***Fig. S1*** Assessment and presentation of the YouTube posts analysed and the respective countries of origin

Out of 89 YouTube posts that were examined, 1 came from Guyana, 12 from the USA, 1 from Japan, 2 from Nepal, 1 from Lebanon, 4 from the United Kingdom, 1 from China, 32 from India, 2 from South Korea, 2 from Malaysia, 6 from Pakistan, 1 from New Zealand, 1 from Uganda, 1 from Germany, 1 from Hungary, 5 from Egypt, 1 from Iraq, 2 from Yemen, 2 from Canada, 1 from Indonesia, 1 from Bangladesh, 1 from Cambodia and 9 were from unknown origins (Fig. S1).


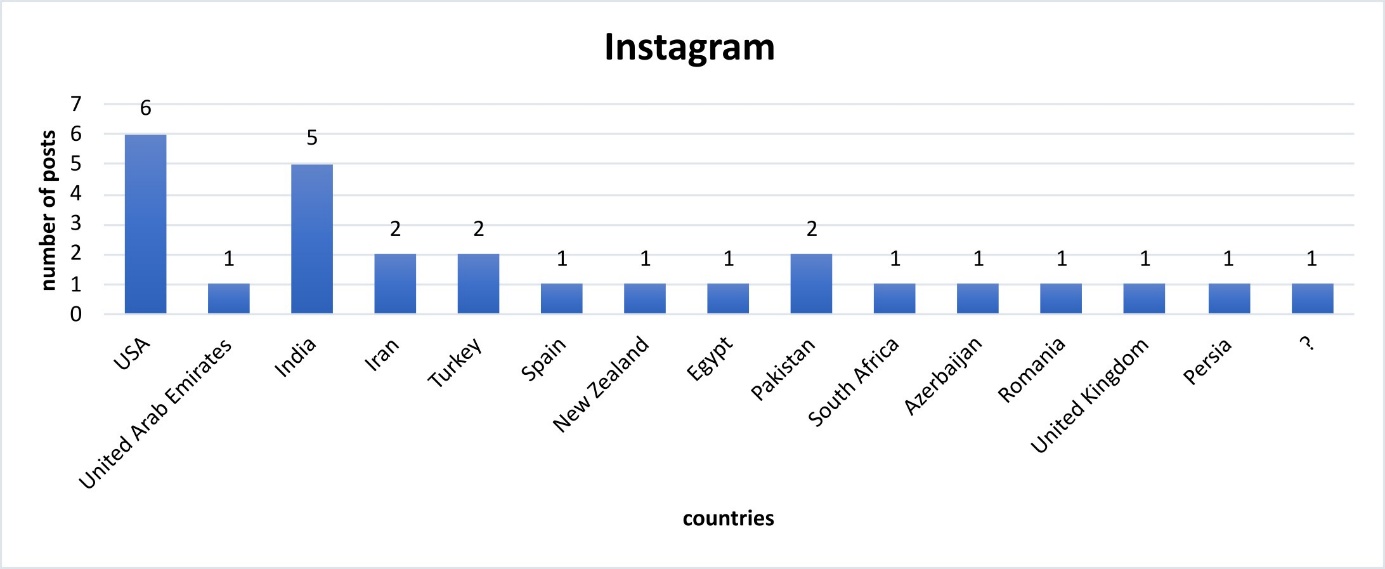
 ***Fig. S2*** Presentation of the Instagram posts analysed and the respective countries of origin

In total 28 Instagram posts were gathered (Fig. S2). 6 posts were from the USA, 1 from the United Arab Emirates, 5 from India, 2 from Iran, 2 from Turkey, 1 from Spain, 1 from New Zealand, 1 from Egypt, 2 from Pakistan, 1 from South Africa, 1 from Azerbaijan, 1 from Romania, 1 from the United Kingdom, 1 from Persia and 1 post came from an unknown origin.


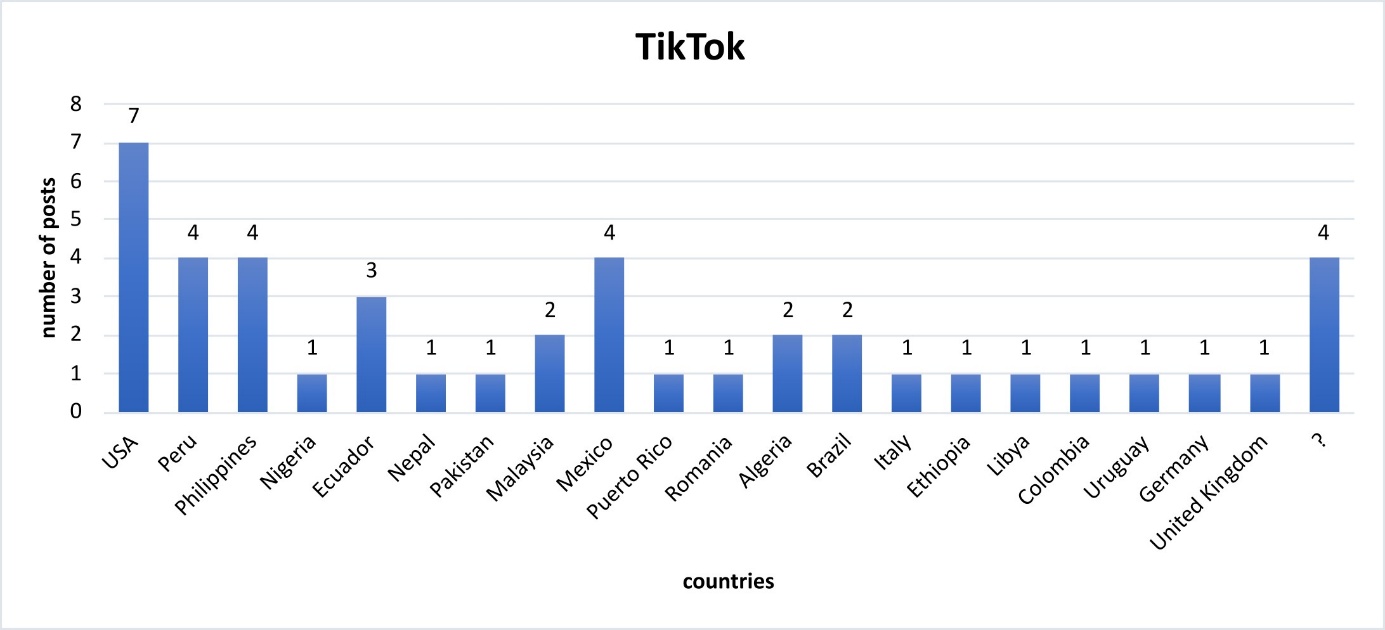
 ***Fig. S3*** Evaluation and presentation of the analysed TikTok posts and the respective countries of origin

In total 44 TikTok posts were examined (Fig. S3). 7 posts were from the USA, 4 from Peru, 4 from the Philippines, 1 from Nigeria, 3 from Ecuador, 1 from Nepal, 1 from Pakistan, 2 from Malaysia, 4 from Mexico, 1 from Puerto Rico, 1 from Romania, 2 from Algeria, 2 from Brazil, 1 from Italy, 1 from Ethiopia, 1 from Libya, 1 from Colombia, 1 from Uruguay, 1 from Germany, 1 from the United Kingdom and 4 from an unknown origin.


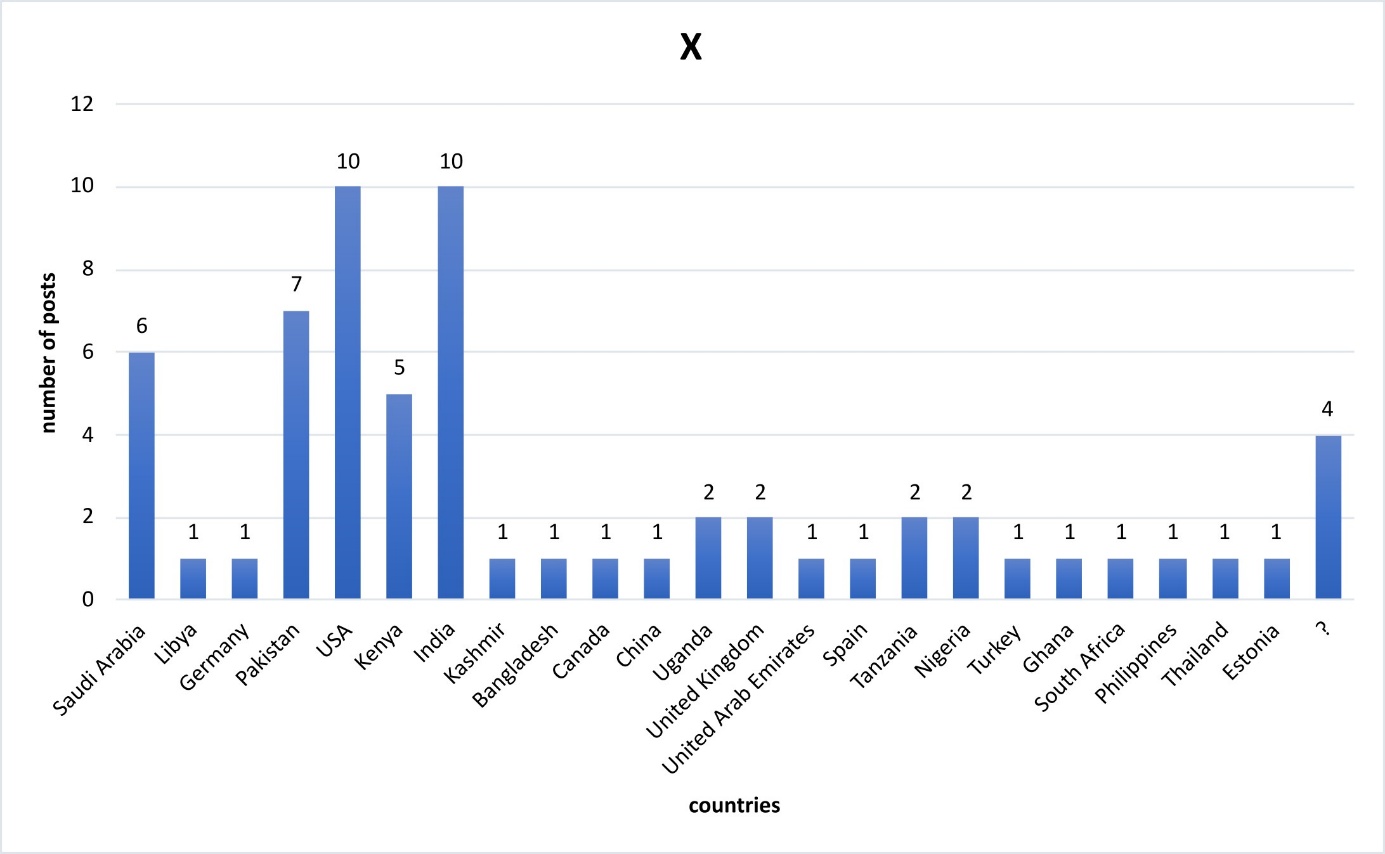
 ***Fig. S4*** Evaluation and presentation of the analysed X posts and the respective countries of origin

In total 64 posts on X were examined (Fig. S4). Out of these 6 posts were from Saudi Arabia, 1 from Libya, 1 from Germany, 7 from Pakistan, 10 from the USA, 5 from Kenya, 10 from India, 1 from Kashmir, 1 from Bangladesh, 1 from Canada, 1 from China, 2 from Uganda, 2 from the United Kingdom, 1 from the United Arab Emirates, 1 from Spain, 2 from Tanzania, 2 from Nigeria, 1 from Turkey, 1 from Ghana, 1from South Africa, 1 from the Philippines, 1 from Thailand, 1 from Estonia and 4 from an unknown origin.
